# How can insulin initiation delivery in a dual-sector health system be optimised? A qualitative study on healthcare professionals’ views

**DOI:** 10.1186/1471-2458-12-313

**Published:** 2012-04-30

**Authors:** Ping Yein Lee, Yew Kong Lee, Chirk Jenn Ng

**Affiliations:** 1 Department of Family Medicine, Faculty of Medicine and Health Sciences, Universiti Putra Malaysia, 43400 UPM Serdang, Selangor, Malaysia; 2 Department of Primary Care Medicine, Faculty of Medicine, University of Malaya, Kuala Lumpur, 58100, Malaysia

**Keywords:** Insulin initiation, Dual-sector health system, Malaysia, Diabetes, Public sector, Private sector

## Abstract

**Background:**

The prevalence of type 2 diabetes is increasing at an alarming rate in developing countries. However, glycaemia control remains suboptimal and insulin use is low. One important barrier is the lack of an efficient and effective insulin initiation delivery approach. This study aimed to document the strategies used and proposed by healthcare professionals to improve insulin initiation in the Malaysian dual-sector (public–private) health system.

**Methods:**

In depth interviews and focus group discussions were conducted in Klang Valley and Seremban, Malaysia in 2010–11. Healthcare professionals consisting of general practitioners (n = 11), medical officers (n = 8), diabetes educators (n = 3), government policy makers (n = 4), family medicine specialists (n = 10) and endocrinologists (n = 2) were interviewed. We used a topic guide to facilitate the interviews, which were audio recorded, transcribed verbatim and analysed using a thematic approach.

**Results:**

Three main themes emerged from the interviews. Firstly, there was a lack of collaboration between the private and public sectors in diabetes care. The general practitioners in the private sector proposed an integrated system for them to refer patients to the public health services for insulin initiation programmes. There could be shared care between the two sectors and this would reduce the disproportionately heavy workload at the public sector. Secondly, besides the support from the government health authority, the healthcare professionals wanted greater involvement of non-government organisations, media and pharmaceutical industry in facilitating insulin initiation in both the public and private sectors. The support included: training of healthcare professionals; developing and disseminating patient education materials; service provision by diabetes education teams; organising programmes for patients’ peer group sessions; increasing awareness and demystifying insulin via public campaigns; and subsidising glucose monitoring equipment. Finally, the healthcare professionals proposed the establishment of multidisciplinary teams as a strategy to increase the rate of insulin initiation. Having team members from different ethnic backgrounds would help to overcome language and cultural differences when communicating with patients.

**Conclusion:**

The challenges faced by a dual-sector health system in delivering insulin initiation may be addressed by greater collaborations between the private and public sectors and governmental and non-government organisations, and among different healthcare professionals.

## Background

The United Kingdom Prospective Diabetes Study (UKPDS) has found that intensification of glycaemic control prevents and delays diabetes-related complications [1]. This often requires oral glucose-lowering drugs in addition to lifestyle modification. However, because of progressive insulin depletion, the majority of patients with type 2 diabetes will require insulin to achieve optimal glycaemic control 5–10 years after diagnosis [1,2]. Internationally, most studies found only about 26–34% of patients with type 2 diabetes achieved optimal glycaemic control [3-6]. This could be related to low employment of an insulin regimen [3,7,8].

In Malaysia, the prevalence of type 2 diabetes ranks seventh in the world [9] and is the highest in the Western Pacific region [10]. A recent study found that 81.9% of Malaysian adults with diabetes seen at the primary care setting did not achieve the recommended glycaemic goal of less than 6.5% haemoglobin A1c [11]. One important reason for poor glycaemic control is the delay in initiating and intensifying insulin therapy [12]. In Malaysia, studies have reported very low usage of insulin among patients with type 2 diabetes [11,13].

Many factors contribute to the delay in insulin initiation in clinical practice: patient factors, such as psychological insulin resistance; clinician factors, such as lack of training and confidence; and system factors. Common system barriers include: short consultation times, rapid staff turnover and lack of continuity of care [14], and these barriers vary across different health systems. Malaysia has a dual-sector healthcare system comprising public (government subsidised) and private (fee for service) sectors. Almost three-quarters of patients with diabetes are managed in the public sector, which often has a high patient load and turnover of doctors [13]. Furthermore, patients in the public sector are often not given a choice of which doctor they would prefer to consult. Therefore, to improve optimisation of glycaemic control and update of insulin, effective and efficient strategies are needed, particularly those targeting the healthcare delivery system. This study aimed to explore the views of Malaysian healthcare professionals (HCPs) on the strategies that would facilitate insulin initiation among patients with type 2 diabetes.

## Methods

### Design

We conducted semi-structured interviews and focus group discussions with HCPs to explore the strategies they used or proposed to improve service delivery in order to facilitate insulin initiation. A qualitative methodology allowed us to explore views on healthcare delivery systems related to the practise of insulin initiation in local practice situations [15]. This also enabled us to have a more holistic view of service delivery and strategies for its improvement [16,17].

HCPs participating in the focus group discussion were grouped according to their practice background and location. This was to ensure homogeneity and to capitalise on shared experiences among the HCPs [18]. For logistic reasons, we conducted individual in-depth interviews with key opinion leaders, such as government policy makers. The use of in-depth interviews, focus group discussions and field notes served to triangulate of the data.

### Setting

In Malaysia, insulin therapy is initiated by HCPs from government hospitals and health clinics; university-based hospitals and primary care clinics; and private hospitals and general practice clinics. In this study, we recruited the HPCs from three states (Wilayah Federal Territory, Negeri Sembilan and Selangor) and from both urban and semi-rural locations. Two key policy makers from the Ministry of Health who were involved in developing and implementing the national diabetes strategic plan were also interviewed.

### Participants, recruitment and sampling

Recruitment was done by researchers. We used purposive sampling to identify the stakeholders who were involved in insulin initiation. They comprised family medicine specialists, general practitioners (GPs), government medical officers and diabetes nurse educators, endocrinologists and government policy makers. We used the ‘snowballing’ technique to recruit participants by asking stakeholders to identify individuals and organisations who were involved in insulin initiation. Some GPs chose not to participate in the study. The reasons were that they were either not interested in research or they perceived that the interviews would disrupt their clinical work as they ran solo practices. We interviewed and analysed in an iterative manner until no new themes emerged. The recruitment was stopped when researchers discussed and reached consensus that the analysis had reached thematic saturation.

### Data collection

An interview topic guide was developed based on literature review and expert opinion. The questions in the topic guide were based on the conceptual framework where health care professionals, patients and the health care delivery system are factors that may influence the initiation of insulin [14]. We interviewed the HCPs using open-ended questions and used prompts only if important issues did not emerge spontaneously during the interview. The HCPs were informed that the interview focused on patients with type 2 diabetes who are indicated to start insulin. The HCPs were asked about the barriers, facilitators and their experience of insulin initiation and this has been reported else [19]. They were also asked to suggest strategies they used or would recommend to optimise insulin initiation. Three trained researchers conducted the individual interviews and focus groups using the topic guide.

We sought written consent from all the participants for audio-recording and the interviews. The participants were informed about the anonymity and the confidentiality of the interview.

An assistant took field notes on non-verbal cues and interview dynamics. Between October, 2010, and May, 2011, we conducted individual interviews and focus groups, lasting about 30 and 60 minutes, respectively. We reached data saturation after ten individual interviews and four focus groups. All the interviews were audio recorded, transcribed verbatim and the transcripts were used as data for analysis.

### Data analysis

A thematic analysis approach was used. The themes were derived inductively from the data. The researchers familiarised themselves with the data by reading and re-reading the transcripts. Three researchers coded two transcripts (interviews with a primary-care physician and a government policy maker) independently and a list of free nodes (themes) was created. The free nodes were merged to form larger categories. This framework, consisting of categories and themes, was used subsequently to code (label) another two transcripts by the researchers independently. The coding was then compared for inter-rater consistency and any discrepancies were resolved by discussion. Attempts were made to reach a consensus, and in the event that disagreement occurred, an independent researcher (who did not participate in the analysis) would be asked to analyse the data separately. However, this situation did not arise in this study. Consensus was reached on the final list of nodes and their descriptions. This final list of revised nodes was imported into Nvivo9 software and served as the framework for coding the rest of the transcripts. New themes that were identified were added to the list upon consultation with the research team. The quotes were chosen based on their representativeness of the themes that emerged from the transcripts. We screened through all the quotes and extracted those that best captured the essence of the themes.

Negative cases were included in the analysis and highlighted to explain why the individual’s views were different from the majority. For example, unlike most GPs, one participant did not have problem initiating insulin with his patients. We explored and realised that he had a special interest in managing diabetes foot and most of his patients presented with complications of diabetes. This highlighted the context of his practice was different from the rest.

Two of the researchers (CJN and PYL) are family medicine specialists and the third is a postgraduate psychologist (YKL). The researchers were conscious of their personal and professional views on insulin initiation. The team underwent constant reflection and open discussion throughout the interviews and analysis to reduce possible biases. This study was part of a larger 3-year study that aimed to develop a patient decision aid for people with type 2 diabetes who are considering insulin therapy.

### Ethics approval

This study received ethics approval from the University of Malaya Medical Centre Medical Ethics Committee and the Medical Research and Ethics Committee of the Ministry of Health, Malaysia.

## Results

### Characteristics of the participants

A total of 38 HCPs participated in the study: 11 general practitioners, ten family medicine specialists, eight medical officers, four government policy makers, three diabetes educators and two endocrinologists. Of the 38 HCPs, 24 were from the government sector and 14 from the private sector. Their mean age was 47 years (range 30–66 years). There were 29 women and nine men; and 13 Malays, 12 Indians, 10 Chinese and three other races.

Three main strategies to improve insulin initiation emerged from the data: (1) collaboration between the public and private sector; (2) greater involvement of pharmaceutical industry, media and non-government organisations (NGOs); and (3) establishment of multidisciplinary teams.

### Collaboration between the public and private sector

Doctors from the private sector lacked resources to initiate insulin. Therefore, HCPs suggested that the government medication subsidies be made available to patients on diabetes follow-up at private facilities. The public sector, on the other hand, faced the problem of a heavy workload and limited consultation time. Shared care between the public and the private sectors would help to overcome the barrier of limited consultation time in the public sector.

"“The Government should set aside a fund, where… it’s not that only poor people should do this thing, I think it should be sort of like… we GPs have no backup. (Government should set aside a fund to be used by private sector GPs for people treated by GPs; currently these GPs have no backup) They should let us, maybe with the patient’s IC (identity card), prove ourselves that our patients are diabetic, and we should at least be able to get the needles and some basic things from the Ministry.” (GP, private practice) "

"“… like my patients I see from the hospitals, they are going to the Klinik Kesihatans (Government Health clinics). All their HbA1c is about 9, 9.5… I don’t blame the doctors, because how much time do they have in contact with the patients? So there should be a sharing treatment on diabetics with private clinics where they should be able to see these doctors and the Government should subsidize their treatment or something, ok in 6 months time you go back here, get your thing, but other times go and visit your nearest clinic, GP clinic, where they can spend more time with you.” (GP, private practice)"

In the private sector, most general practitioners did not have supporting staff such as diabetic educators and dieticians to provide patient education. One option was to enrol their patients for diabetes education in the government health clinics.

"“I mean the Government has a lot of budget for many mega-projects, … and I think there should be some kind of subsidy (patient education) program when GPs can participate. … It shouldn’t just be limited to the hospital. The participation should be open to the GPs.” (GP, private practice)"

### Greater involvement of pharmaceutical industry, media and non-government organisations

#### Pharmaceutical industry

Pharmaceutical companies could play an important role in continuing medical education by organising training and workshops on insulin initiation for HCPs.

"“So, that is actually with the help of some educational grant, there’s been a lot of help from the pharma industry...they have also given a grant to run the workshops …” (Endocrinologist, government hospital)"

Pharmaceutical representatives may act as a resource person to support the doctors in starting their patients on insulin. Unlike medical colleagues, the pharmaceutical representatives spent time with the GPs and guided them through the insulin initiation process step-by-step.

"“The pharma companies, they have been very good, so the guy will come with every literature to me, he will train me up, and then I will say, oh, so many units, what if the patient goes into hypo (hypoglycaemia), and then they will have to convince me, doctor, they are not going to go into hypo. We believe in that because we are going to start with a very low dose, these drugs are very different from the old insulin. So they are holding my hand, and they have guided me to use insulin...so I have learnt my insulin not through any endocrinologist, not through any doctor, but these guys… they walk the talk, walk the talk with me.” (GP, private practice)"

In the government sector, pharmaceutical companies were involved in providing patient educational and decision support material, which healthcare providers used when helping a patient to make decisions about initiating insulin. Most of the patient health education materials on insulin were developed and provided by the pharmaceutical companies.

"“Decision maps like those provided by Pharmaceutical Company A, that kind of thing. Some clinics have started doing that, erm… it is something which we supported, but I’m not sure how many clinics are motivated enough to move, to want to organise. …I see it as a good tool. I see it as a different approach to health education.” (Government policy maker)"

"“That’s not the ‘in’ thing in Malaysia. Pharma. A lot people are not comfortable. For me, as long as it’s not biased. You know. This one (guide book) for example, this is supported… printing supported by Pharmaceutical Company B…(I) have to source for the fund. The ministry don’t want to pay. I think, that it’s a bit ridiculous. I asked Pharmaceutical Company B, I asked around, I asked Pharmaceutical Company C, Company C don’t want.” (Government policy maker)"

"“So, that is actually with the help of some educational grant, there’s been a lot of help from the pharma industry, so even to develop this guide, it is actually with the help of pharma…” (Endocrinologist, government hospital)"

The pharmaceutical industry also helped to supply insulin pens to patients free of charge. In addition, in the private sector, where there was a lack of resources, some pharmaceutical companies employed diabetes educators to assist the doctors in educating patients about insulin therapy.

"“For me I would talk to the company and tell them, you make sure if you want me to use your insulin, you had better supply enough pens for me.” (Family Medicine Specialist, Government health clinic)"

"“You can get them (pharmaceutical companies)…you can just give them a call, and you have a certain patient you think has to be on insulin, and that patient refuses to take the insulin in spite you have informed him, and you find resistance. And you can get these people, these people who market you this insulin, they will do the marketing for you. They will go to the house; they’ll talk to the patient. I had one patient who had a problem, but after about 5 months the patient finally accepted to take insulin. (GP, private practice)"

#### Media

Some HCPs felt that the media played an important role in educating the public about diabetes and the benefits of insulin.

"“I feel the media should play a part. An important role. Like, you know, a TV channel… Just every day, two, three times say, 5 minutes, what is diabetes, how important it is, how insulin is important… Because every day we all see the TV, in Tamil, or Malay, or Chinese, or English… if they put every day 5 minutes of time out, three times a day, I think people will think, you know, these are the psychological feelings, every day goes to the mind and they come to think of it.” (GP, private practice)"

#### Non-government organisations

NGOs could be involved in organisation of conferences for training of HCPs in education and counselling of patients with diabetes.

"“…health counselling or health education delivery. Erm… Prof Prochaska’s, transtheoretical model, he came to Malaysia last year. So, there was a diabetes conference, erm… held by the erm, the Diabetes Education arm of, educators arm, of Persatuan Diabetes Malaysia (Malaysia Diabetes Association) ….” (Government policy maker)"

When facing time constraints and lack of expertise, some doctors from the private sector felt that NGOs could help by providing a dietetic service and by engaging diabetes educators to counsel patients about insulin therapy.

"“…behind (my clinic) there’s diabetic centre of Malaysia… The diabetes centre is just behind. So if they actually need some further explanation, just go round the corner …that’s for my area. And then there’s also the nutrition specialist, they have full diet (full dietary advice) and everything they will…can be referred. So in our locality, quite easy.” (GP, private practice)"

Other important roles of NGOs included organising peer support group sessions, health screening and road shows; and subsidising glucometers, strips and needles for patients with diabetes.

"“We have two big NGOs for diabetes, which is um…the PDM, Malaysian Diabetes Association and then NADI, the National Diabetes Institute. So in terms of patient support, um…you know I mean…PDM is good in the sense that it gives patients the facilities to get uh…you know…I mean, at cost price all the equipment, test strips, meters and…and they have very good network, branches all over the country. So, they’re actually helping patients. …they are developing patient support material and then they go for road shows for screening, public screening, the usual thing.” (Endocrinologist, government hospital)"

### Multidisciplinary team

The HCPs suggested that setting up multidisciplinary teams, consisting of doctors, assistant medical officers, diabetic educators, nurses, pharmacists and dieticians, would greatly facilitate insulin initiation. However, information provision should be consistent to avoid giving contradictory advice. The involvement of other healthcare team members could overcome the time constraints of doctors to counsel patients.

"“…ermm, forming a multidisciplinary team. Although probably not a complete team like they have in the hospital, but at least you should have a nurse, a medical officer, a specialist will not be available in our clinic. And then the pharmacist actually can be involved in the team so that everybody should be having a role and then of course you have to make sure that these people understand each others’ roles and are giving similar information.” (Family Medicine Specialist, government health clinic)"

"“Uh, in the way there’s short of time, the patient lack of counselling, so I have to get somebody, the counselling nurse, to do the counselling. So those are uncontrolled they will send to the nurse, the nurse will try to talk to the patient and talk about diet, exercise, all those stuff. And then uh they will go back and take their medicine and go. So, with the counselling nurse on and then subsequently the pharmacists, uh, it can improve a bit. I think for the doctor it’s very difficult for the MOs (medical officers) to do the talking. They have no time to talk, basically.” (Family Medicine Specialist, government health clinic)"

To overcome the problem of short consultation time and fast turnover of doctors in the government clinics, policy makers advocated the empowerment of paramedical staff to counsel patients with diabetes who needed insulin. The proposed strategies included a reference guide and training programmes targeted at the allied health workers.

"“Policy level, I feel, although the paramedics can’t prescribe, I feel that they actually can play a big role in influencing patient's decision, whether they want it or not, how empowered are they to… to… self-titrate, or to monitor. Erm, at the primary care level, because they are the constant figure in that particular clinic, the doctors come and go. So, I’m keen actually, for this, for example, for the insulin to come out with the quick reference to teach the paramedics as well. These are the things that can be done.” (Government policy maker)"

The HCPs emphasised the importance of teamwork in helping patients to control their diabetes and to advise them on insulin initiation.

"“Hmm… so that’s why our arrangement there… before the doctor sometimes sees us first, then we educate them…ha… so we explain to them what they should do, should increase which medicine, why sugar levels are high, why sugar levels are low, what…hmm… things like that… so teamwork is always better.” (Diabetic educator, government university hospital)"

"“It should be…there must be a diabetic educator, dietician. It should be a combined work, not only a doctor who does this. There must be team play. A team work to do this.” (GP, private practice)"

Some doctors noted that having team members from different ethnic backgrounds helped to overcome the language barriers they face during consultations.

"“…that uh language, I feel, is a very important barrier you have to overcome. But anyhow with the help of my MOH (Medical officer of Health)I manage to get, uh, lots of Indian staff to be in the clinic. Even I got the sister who’s Indian, I got the attendant who is Indian. So, basically it when we improve the communication, the patient can accept it (the treatment) better.” (Family Medicine Specialist, government health clinic)"

## Discussion

The finding from the study highlighted three main strategies to improve insulin initiation in a dual-sector health system: (1) collaboration between the public and private sector; (2) greater involvement of pharmaceutical industry, media and non-government organisations (NGOs); and (3) establishment of multidisciplinary teams.

The participants highlighted the uneven distribution of resources for the management of chronic diseases such as diabetes within the dual-sector healthcare system. The government health clinics are facing a shortage of doctors [20-22], and the rising incidence of type 2 diabetes will aggravate the situation as the majority of patients with diabetes are managed in the public health sector. On the other hand, most of the private GPs in Malaysia run solo practices and they lack resources and support to initiate insulin. The lack of integration and collaboration of the dual-sector health system is a major barrier for insulin initiation in patients with diabetes. However, this dual-sector healthcare system may provide a good opportunity to improve the care of diabetes by utilising the strength of each sector to integrate diabetic care. Studies by the World Health Organisation and others have found that an integrated health system can be effective in improving quality of care [23,24]. Recently, a new national healthcare financing mechanism has been proposed to integrate the public and private healthcare systems under the 9^th^ Malaysia Plan 2006–2010, and this includes the primary care services [25-27]. This would help to reduce the existing discrepancy in the distribution of resources and manpower between the public and private sectors for diabetes care, as emphasised by the HCPs in this study.

In this study, the HPCs also highlighted the role of the pharmaceutical industry in providing HCP training and diabetes educators to counsel patients. In recent years, pharmaceutical companies have faced criticism [28-30] and there are rising concerns about their influence on the HCPs’ prescribing decisions [31,32]. Restricting contacts between the pharmaceutical industry and HCPs could limit open dialogue, hamper innovation and create a gap in educational support for HCPs, at least in developing countries like Malaysia [33]. Moreover, collaboration may result in mutual benefit for all parties, including health professionals, the pharmaceutical industry and patients [34]. In the care of diabetes and insulin initiation, the collaboration between HCPs and the pharmaceutical industry in educational programmes and counselling for patients will eventually benefit all parties. However, some regulations are needed to prevent undue influence from the pharmaceutical companies. Some countries have put in place processes, such as the review and management of research, industry codes of conduct, community responses and guidelines by practitioner associations, to protect the interests of individual patients and community interests [35-38]. This may also help to foster research and the development of new products, maintain public confidence in pharmaceuticals and medicine, and facilitate ethical decision making among various stakeholders [35,39,40]. The Malaysian Government and local professional bodies in the country may need to develop more comprehensive regulations in relation to the involvement of pharmaceutical companies in supporting health promotion programmes.

Non-profit, non-government health organisations play an important role in providing counselling services and support in terms of health education, peer group programmes and financial assistance to patients with diabetes who need insulin initiation. Danika et al. reported that non-profit organisation-sponsored programmes promoting awareness about a disease or health condition are more effective than those sponsored by a pharmaceutical company [41]. Some consumer groups have stressed the importance of active collaborations between health consumer organisations and the pharmaceutical industry [42].

The multidisciplinary team approach to diabetes care, such as insulin initiation, is considered an essential step towards improvement of patient care [43,44]. Besides improving the efficiency of the diabetes service by reducing the doctor’s consultation time, a multidisciplinary team has been shown to improve glycaemic control, lower the risk of diabetes complications, decrease health care costs and improve patients’ quality of life [45,46]. As highlighted by the HCPs in this study, a multidisciplinary team from different ethnic backgrounds is crucial to overcome the problem of language and cultural barriers, particularly in a multi-ethnic country like Malaysia. In a review by Caballero, increased cultural awareness and use of diabetes educators speaking the same language as the patients improved acceptance of insulin therapy in patients with type 2 diabetes [47]. Currently, in Malaysia, the diabetes care teams are located mainly in the government health care clinics or university primary care clinics in urban areas. In the rural settings, many patients with diabetes are managed by medical assistants, whose role is mainly to prescribe, provide basic health education and identify complications. In view of the benefits of a multidisciplinary team approach, Malaysia’s healthcare system should empower the allied health workers, such as the medical assistants, by continuously training and enhancing their knowledge and skills on diabetes care, including insulin initiation. This will reduce the healthcare burden and cost without compromising patient care.

The strength of this study lies in the fact that the sample encompassed all healthcare sectors and stakeholders who were involved in insulin initiation. We were thus able to gather data from all levels of HCPs involved in diabetes care. . This study also allows comparison of views from the private and government HCPs in a dual-sector health system, which may be applicable to other developing countries with a similar health system.

The limitation of this study was that only HCPs’ perspectives were included and patients’ views were not captured. The researchers are planning to conduct interviews with patients with type 2 diabetes who are considering insulin as part of a larger study. Besides that, as the study was conducted in urban and semi-rural areas, therefore the findings cannot be generalisable to rural settings. Finally, two of the researchers are primary care physicians (LPY and NCJ) and this may influence the interpretation of the data. These potential biases are reduced by constant reflection by the two researchers about their roles and by involving an independent non-clinician (LYK) in the analysis process.

Future research should look into how pharmaceutical industry may be involved in educating the HCPs in the use of insulin especially in resource-limited countries. Secondly, the policy makers should develop strategies to facilitate collaborations between public and private health sectors especially in terms of how resources can be shared more effectively.

## Conclusions

The importance of integration and collaboration between the public and private sectors, multidisciplinary teamwork and active involvement of NGOs was considered as crucial to improve service delivery for insulin initiation and diabetes care in Malaysia. The involvement of pharmaceutical industry and NGOs may be important in the resource-limited private sector. However, some regulations need to put in place to prevent undue influence from the pharmaceutical industries on physicians’ clinical decisions. Therefore, a proposed integration of the public and private healthcare systems may help to make diabetes care, including insulin initiation delivery, more effective and efficient.

## Abbreviations

GP: General practitioner; HCP: Healthcare professional; NGO: Non-government organisation; UKPDS: United Kingdom Prospective Diabetes Study.

## Competing interests

The authors declare that they have no competing interests.

## Authors’ contributions

PYL wrote the first draft of the paper and led the critical review and revision of the paper. PYL, YKL and CJN were involved in the study design, development of the interview topic guides used for data collection, facilitation of the data collection interviews and focus groups, analysis of the data for reporting, interpretation of the data, and review of the paper. All authors have read and approved the final manuscript.

## Pre-publication history

The pre-publication history for this paper can be accessed here:

http://www.biomedcentral.com/1471-2458/12/313/prepub
